# Highlights of light meson spectroscopy at the BESIII experiment

**DOI:** 10.1093/nsr/nwab198

**Published:** 2021-11-02

**Authors:** Shan Jin, Xiaoyan Shen

**Affiliations:** School of Physics, Nanjing University, Nanjing 210008, China; Institute of High Energy Physics, Chinese Academy of Sciences, Beijing 100049, China; University of Chinese Academy of Sciences, Beijing 100049, China

**Keywords:** hadron, spectroscopy, charmonium, meson, glueball, hybrid, multi-quark state

## Abstract

Hadron spectroscopy provides a way to understand the dynamics of the strong interaction. For light hadron systems, only phenomenological models or lattice quantum chromodynamics (QCD) are applicable, because of the failure of perturbation expansions for QCD at low energy. Experimental data on light hadron spectroscopy are therefore crucial to provide necessary constraints on various theoretical models. Light meson spectroscopy has been studied using charmonium decays with the Beijing Spectrometer Experiment (BES) at the Beijing Electron-Positron Collider, operating at 2.0–4.6 GeV center-of-mass energy, for nearly three decades. Charmonium data with unprecedented statistics and well-defined initial and final states provide BESIII with unique opportunities to search for glueballs, hybrids and multi-quark states, as well as perform systematic studies of the properties of conventional light mesons. In this article, we review BESIII results that address these issues.

## Teaser

We review BESIII results on the study of glueballs and multi-quark states, which shed light on the understanding of the strong interaction.

## INTRODUCTION

Our knowledge of mesons and, in parallel, our understanding of the strong interactions have undergone several major revisions. Mesons were first introduced when Yukawa [1] predicted the existence of pions as the exchange boson responsible for the strong interaction between nucleons. Later, more and more mesons and baryons showed up in cosmic ray and high-energy accelerator experiments. It was eventually realized that light hadrons, mesons and baryons of a given *J*^*PC*^ are arranged in representations of the SU(3) group, and this led to the quark model by Gell-Man and Zweig [2,3]. In the quark model, hadrons are, in fact, objects that are comprised of constituent spin-$\frac{1}{2}$ fermions, called quarks. Constituent quarks are valence quarks for which the correlations for the description of hadrons by means of gluons and sea quarks are put into effective quark masses of these valence quarks. Mesons are made of quark-antiquark ($q \bar{q}$) pairs and baryons are made of three quark (*qqq*) combinations. With this simple quark scheme, the qualitative properties of hadrons were explained quite well. However, serious problems with the Pauli exclusion principle occurred for some of the quark wavefunctions. This problem was solved when Greenberg [4] pointed out that quarks had another quantum number that was subsequently named ‘color’. But still, considerable skepticism about the quark model persisted, primarily due to the fact that isolated quarks were never observed. This situation changed when the results from deep inelastic scattering of electrons on protons and bound neutrons [5] came out in 1968, indicating the presence of hard and point-like components in neucleons, and the discovery of *J*/ψ was reported in 1974 [6,7], which was interpreted as the bound state of a new heavy quark ‘charm’ and its antiquark, as proposed by Glashow *et al.* [8]. Subsequent experimental and theoretical developments proved to be convincing evidence that quarks were real objects and the fundamental building blocks of hadronic matter.

The constituent quark model (CQM) proposed by Gell-Mann and Zweig was able to reproduce the charmonium spectrum and describe the phenomenology of meson and baryon spectroscopy rather well. However, problems remained. The well-accepted theory of the strong interaction is quantum chromodynamics (QCD) [9,10], a non-Abelian gauge-field theory that describes the interactions of quarks and gluons and has the features of asymptotic freedom and confinement of quarks. For the light scalars, such as *f*_0_(500), the dispersive formalisms, which are shown to follow from first principles, determine the mass and width of *f*_0_(500) within small uncertainties [11,12]. For the mesons containing at least one heavy (*c* or *b*) quark, the simulations using non-relativistic QCD or heavy quark effective theory, which expanses the QCD Lagrangian in powers of the heavy quark velocity, or the heavy quark mass, have become a high-precision task [13–15]. However, first-principle computations, directly from the QCD Lagrangian, of hadron properties for light hadrons are difficult, due to the failure of perturbation expansions for QCD at low energies. As a result, our knowledge of light hadrons mainly relies on either QCD-based phenomenological approaches or lattice QCD (LQCD) calculations. Lattice calculations of QCD are a major source of information about QCD masses and matrix elements. A review of the hadron spectrum from lattice QCD can be found in the review of the quark model in Particle Data Group 2020 (PDG 2020) [16] and in [17]. Aside from the conventional $q \bar{q}$ mesons and *qqq* baryons in the CQM, QCD-based models also allow the possible existence of bound states that are made of only gluons, i.e. so-called ‘glueballs’. Furthermore, it is also possible to form multi-quark hadrons, with the number of quarks larger than three, and ‘hybrids’ that contain both $q\bar{q}$/*qqq* and at least one gluon (*g*) as its constituents, $q\bar{q}g$/*qqqg*. All of these unconventional states, so-called new forms of hadrons, if they exist, will greatly enrich the spectra of mesons and baryons, and shed light on the dynamics of long-distance QCD. In the past decades, despite the fact that LQCD has experienced dramatic improvements together with rapid developments in computing resources, there still remain many technical difficulties in the extraction of precise properties of glueballs, hybrids and multi-quark states in LQCD calculations. Moreover, these new forms of hadrons may have *J*^*PC*^ that are the same as those of CQM states and thus mix with conventional hadrons, which makes their identification more complicated. Still, searching for unconventional hadrons, such as glueballs, hybrids and multi-quark states, as well as investigating their spectra in experiments have been important subjects of modern (intermediate) high-energy physics for many decades. In particular, the observations of new hadron candidates, XYZ states with heavy quarks in the past decade, have drawn further attention in this field.

Many experiments have been dedicated to studies of light hadron spectroscopy. In recent years, charmonium data samples with unprecedent statistics were accumulated by the Beijing Spectrometer (BESIII) at the Beijing Electro-Positron Collider (BEPCII), and these provide numerous opportunities for investigating light hadrons produced in charmonium decays. In this article, the highlights on studies of glueball and hybrid candidates, and searches for the multi-quark states from *J*/ψ decays by the BESIII experiment are reviewed.

## STUDY OF THE GLUEBALL CANDIDATES

The CQM has had considerable success in predicting the spectrum of hadrons and their decay properties. However, CQM is only a phenomenological model, and it is not derived from the underlying QCD theory of the strong interaction. Therefore, the CQM spectrum is not necessarily the same as the physical spectrum in the QCD theory. QCD-based phenomenological models, such as bag models [18–21], flux-tube models [22,23], QCD sum rules [24–27] and LQCD [28–31] can make predictions of the masses and other properties of glueballs. Of them, the only first-principle calculations of spectroscopy from QCD is LQCD.

**Figure 1.**
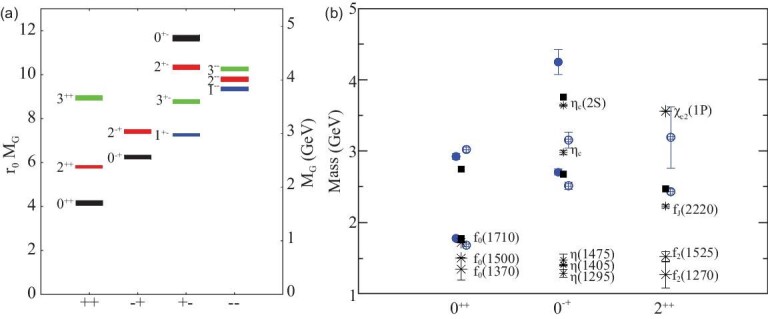
(a) The mass spectrum of the lightest glueball states predicted from quenched LQCD calculations [31]. (b) The mass spectrum of glueball states predicted from unquenched LQCD [32]. The open and filled circles are the full QCD calculation of glueball masses, with larger and smaller lattice spacing, respectively. Squares are the quenched calculations for glueball masses of [30]. The bursts labeled by particle names are experimental states.

In the early years, most of the LQCD calculations of the glueball spectrum were confined to the quenched approximation in which internal quark loops are neglected. Figure 1(a) shows the results of the glueball spectrum for the lightest glueballs from quenched calculations [31]. The lowest glueball is a *J*^*PC*^ = 0^++^ scalar state with mass in the range 1.5–1.7 GeV/c^2^; the next lightest glueball is a *J*^*PC*^ = 2^++^ tensor state with mass around 2.4 GeV/c^2^. For the lightest *J*^*PC*^ = 0^−+^ pseudoscalar glueball, the LQCD calculated mass is above 2.3 GeV/c^2^. In recent years, the lattice calculations of the glueball spectrum with dynamical light quarks and high statistics appeared [32,33]. Figure 1(b) shows the unquenched results for the glueball spectrum from [32], along with comparisons to the quenched lattice calculation of [30] and to experimental isosinglet mesons. The effects of quenching seem to be small, and the quenched and unquenched predicted masses for the lightest glueballs are close to each other.

As for glueball couplings and decay rates, we still lack first-principle theoretical predictions, although some expectations [34] from phenomenological models for a glueball with conventional quantum numbers can provide useful guidance for distinguishing a glueball candidate from a conventional hadron, such as

- well-established states that lack a close correspondence or a clear assignment to quark model nonets;
- enhanced production in gluon-rich processes, such as *J*/ψ radiative decays, *pp* central productions and $p \bar{p}$ annihilations;
- flavor blindness of glueball decays—since glueballs are SU(3) flavor singlets, they are expected to couple equally to *u*, *d* and *s* quarks;
- the production of glueballs in two-photon collisions and the decay of glueballs into two-photon final states are expected to be suppressed, since gluons are electrically neutral.

On the other hand, the properties of glueballs are not expected to be significantly different from those of conventional hadrons. These make the identification of a glueball more complicated and difficult.

### The scalar and tensor glueball candidates

The scalar and tensor meson spectra have been studied in many reactions, including pion induced reactions π^−^*p* [35], $p \bar{p}$ annihilations [36–39], central *pp* collisions [40–42] and the decays of ψ(2*S*) [43], *J*/ψ [44–52], *B* [53], *D* [54], φ [55] and *K* [56] mesons, as well as two-photon processes [57]. An attractive and important feature in the study of two-pseudoscalar systems, such as ππ, $K \bar{K}$ and ηη, in radiative *J*/ψ decays is the simplicity in the partial wave analysis (PWA), a generally accepted method of amplitude analysis to determine the spin parities of intermediate states in decay processes. Conservation of parity in strong and electromagnetic interactions, as well as the conservation of angular momentum, restrict the quantum numbers of the pseudoscalar-pseudoscalar pairs. Thus, for pseudoscalar pairs produced in *J*/ψ radiative decays, only amplitudes with even angular momentum and positive parity and charge conjugation quantum numbers are accessible (*J*^*PC*^ = 0^++^, 2^++^, 4^++^, etc.). While in the two-vector systems (φφ, ωω, etc.) in *J*/ψ radiative decays, pseudoscalar, scalar and tensor mesons with the masses higher than 2 GeV/c^2^ can be accessed.

The scalar resonances *f*_0_(1500) and *f*_0_(1710) are main competitors for the lightest 0^++^ glueball candidates, since they are copiously produced in gluon-rich processes and both have masses that are near the LQCD predicted values. The inclusion of data from radiative *J*/ψ decays provides a source that is complementary to hadronic production experiments.

**Figure 2.**
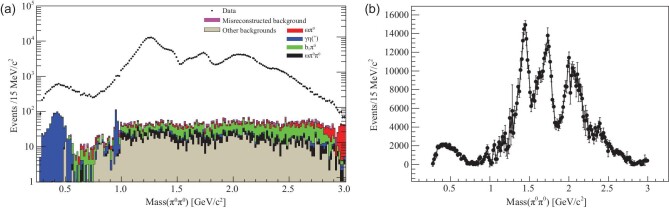
(a) The $M_{\pi ^{0}\pi ^{0}}$ invariant mass spectrum after all selection criteria have been applied. The black markers represent the data, while the histograms are the backgrounds from Monte Carlo simulations. (b) The PWA-determined intensities for the 0^++^ as a function of $M_{\pi ^{0}\pi ^{0}}$ (only statistical errors are presented).

Radiative *J*/ψ decays to π^+^π^−^ and π^0^π^0^ have been studied by the MARKIII [44], DM2 [45], Crystal Ball [46] and BES [58,59] experiments. Based on a sample of 1.3 × 10^9^* J*/ψ events accumulated with the BESIII detector [60], *J*/ψ → γπ^0^π^0^ decays [58] were used to study *f*_0_(1500) and *f*_0_(1710). The π^0^π^0^ invariant mass spectrum for the selected *J*/ψ → γπ^0^π^0^ events is shown in Fig. 2(a) as the black dots with error bars. A strong well-known *f*_2_(1270) signal, a shoulder on the high mass side of *f*_2_(1270), an enhancement at ∼1.7 GeV/c^2^ and a peak at ∼ 2.1 GeV/c^2^ are evident. A mass-independent PWA was performed, where the amplitudes for radiative *J*/ψ decays to π^0^π^0^ are constructed in the radiative multipole basis, as described in detail in Appendix A of [58]. The components of the ππ amplitude were measured independently for many ππ invariant mass intervals. This provides a piecewise complex function that describes the ππ dynamics with minimal assumptions. Figure 2(b) shows the intensities for the 0^++^ amplitudes as a function of $M_{\pi ^{0}\pi ^{0}}$ that are determined by the mass-independent PWA, where there are significant 0^++^ structures just below 1.5 GeV/c^2^ and near 1.7 GeV/c^2^. In the mass-dependent PWA, the *s* dependence of the ππ interaction (where *s* is the invariant mass squared of the two pions) is parameterized as a coherent sum of resonances, each described by a Breit–Wigner line shape with resonance properties, e.g. the mass, width and branching fraction, that are extracted from the fit. The preceding BESII experiment [61] performed a mass-dependent PWA in *J*/ψ → γπ^+^π^−^ and γπ^0^π^0^, using relativistic covariant tensor amplitudes constructed from Lorentz-invariant combinations of the polarization and four-momentum vectors of the initial- and final-state particles, with helicity ±1 *J*/ψ initial states [62]. The PWA results [59] show similar features as those extracted from the BESIII mass-independent PWA [58]. The measured product branching fractions for *f*_0_(1500) and *f*_0_(1710) decaying to ππ are listed in Table 1.

**Table 1.** The product branching fractions for ${\cal B}(J/\psi \rightarrow \gamma X) \times {\cal B}(X \rightarrow M_1 M_2)$ in different decay channels.

			
	*f*_0_(1500)	*f*_0_(1710)	*f*_2_(2340)
	(10^−5^)	(10^−5^)	(10^−5^)
ππ	1.01 ± 0.30	4.00 ± 1.00	
$K \bar{K}$	$6.36 \pm 0.64 \, {^{+\, 0.72}_{-\, 2.24}}$	$80.00\, ^{+\, 1.20}_{-\, 0.80}\, {^{+\, 1.20}_{-\, 4.00}}$	$5.54 \, ^{+\, 0.34}_{-\, 0.40}\, {^{+\, 3.82}_{-\, 1.49}}$
ηη	$1.65\, ^{+\, 0.26}_{-\, 0.31}\, {^{+\, 0.51}_{-\, 1.40}}$	$23.50\, ^{+\, 1.30}_{-\, 1.10}\, {^{+\, 12.40}_{-\, 7.40}}$	$5.60\, ^{+\, 0.62}_{-\, 0.65}\, {^{+\, 2.37}_{-\, 2.07}}$
φφ			$1.91 \pm 0.14\, ^{+\, 0.72}_{-\, 0.73}$

**Figure 3.**
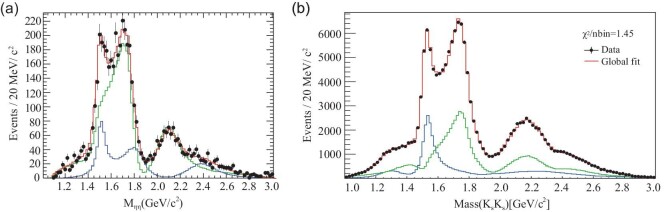
(a) The ηη invariant mass distributions for the selected γηη candidates. (b) The *K*_*S*_*K*_*S*_ invariant mass distributions for the selected γ*K*_*S*_*K*_*S*_ candidates. In both plots, the black points with error bars are data, the green and blue histograms are contributions from the 0^++^ and 2^++^, respectively, and the red histogram is the PWA fit projection of all contributions.

Scalar and tensor glueball candidates were also studied with *J*/ψ radiative decays to ηη and $K \bar{K}$. Using 2.25 × 10^8^ *J*/ψ events collected with the BESIII detector, the decays of *J*/ψ → γηη were investigated [63]. The black points with error bars in Fig. 3(a) show the invariant mass distributions of ηη for the selected γηη candidates, where peaks around 1.5, 1.7 and 2.1 GeV/c^2^ are apparent. A mass-dependent PWA was carried out, and the results indicate that the peak at around 1.5 GeV/c^2^ is mainly from the well-established tensor state $f_2^{\prime }(1525)$ with some contribution from *f*_0_(1500). The statistical significance of the *f*_0_(1500) signal is 8σ. The peaks around 1.7 and 2.1 GeV/c^2^ are dominated by *f*_0_(1710) and *f*_0_(2100), respectively, and the significance for the presence of a tensor *f*_2_(2340) state is 7.6 σ. The red histogram in Fig. 3(a) shows the PWA fit projection with all of the components included, which agrees well with data. The green and blue histograms in Fig. 3(a) represent the contributions from 0^++^ and 2^++^ components, respectively.

A study of the *K*_*S*_*K*_*S*_ system produced in radiative *J*/ψ decays was performed [64] using 1.3 × 10^9^ *J*/ψ decays collected by the BESIII detector. The black dots with error bars in Fig. 3(b) show the invariant mass spectrum of *K*_*S*_*K*_*S*_ for the selected γ*K*_*S*_*K*_*S*_ events. Three significant peaks in the *K*_*S*_*K*_*S*_ mass spectrum around 1.5, 1.7 and 2.2 GeV/c^2^ are observed. A mass-dependent amplitude analysis was applied to extract the parameters and product branching fractions of the resonances that parameterized the *K*_*S*_*K*_*S*_ invariant mass spectrum as a sum of Breit–Wigner line shapes. In addition, a mass-independent analysis was performed to obtain the function that describes the dynamics of the *K*_*S*_*K*_*S*_ system while making minimal assumptions about the properties and number of poles in the amplitudes. The two approaches give consistent results. The red histogram in Fig. 3(b) shows the PWA fit projection for all components, which agrees well with data. The green and blue histograms represent the contributions from the 0^++^ and 2^++^ components, respectively. The dominant scalar contributions come from *f*_0_(1500), *f*_0_(1710), and *f*_0_(2200). The tensor spectrum in *J*/ψ → γ*K*_*S*_*K*_*S*_ is dominated by the well-known $f_{2}^\prime (1525)$. However, an additional *f*_2_(2340) is needed in the fit.

The measured product branching fractions for the *f*_0_(1500) and *f*_0_(1710) scalars and the *f*_2_(2340) tensor in *J*/ψ → γηη and γ*K*_*S*_*K*_*S*_ are listed in Table 1. In both decay modes, the product branching fractions for *f*_0_(1710) are about an order of magnitude larger than that for *f*_0_(1500). A contribution from *f*_2_(2340) is needed in both the *J*/ψ → γηη and *J*/ψ → γ*K*_*S*_*K*_*S*_ channels. The mass of the tensor state *f*_2_(2340) is consistent with the LQCD prediction for a pure tensor glueball.

**Figure 4.**
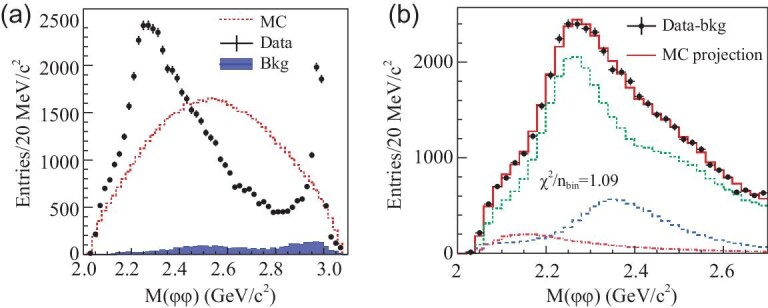
(a) and (b) The φφ invariant mass distributions for the selected γφφ candidates. The black points with error bars are data. The red short-dashed histogram in (a) shows the phase space shape from a Monte Carlo (MC) simulation. The green short-dashed, the red dash–dot and the blue long-dashed histograms in (b) are the coherent superpositions of the Breit-Wigner (BW) resonances with *J*^*PC*^ = 0^−+^, 0^++^ and 2^++^, respectively, and the red solid histogram in (b) shows the total contribution from all components.

The φφ invariant mass distribution for selected radiative *J*/ψ → γφφ decay events [65], from the same 1.3 × 10^9^ *J*/ψ data sample, is shown as black dots with error bars in Fig. 4(a). A distinct η_*c*_ signal and clear structures at lower φφ invariant masses are observed. Both mass-dependent and mass-independent PWA were performed for the *M*(φφ) < 2.7 GeV/c^2^ region with results that are consistent. In addition to three dominant 0^−+^ pseudoscalar states η(2225), η(2100) and *X*(2500), three tensors, *f*_2_(2010), *f*_2_(2300) and *f*_2_(2340), and one scalar *f*_0_(2100) contribute significantly in the PWA fit. The green short-dashed, the red dash–dot and the blue long-dashed histograms in Fig. 4(b) show the coherent superpositions of the Breit–Wigner resonances with *J*^*PC*^ = 0^−+^, 0^++^ and 2^++^, respectively from the model-dependent PWA fit, and the red solid histogram shows the total contribution from all components, which is in good agreement with data. The statistical significance of *f*_2_(2340) → φφ is 11σ.

**Figure 5.**
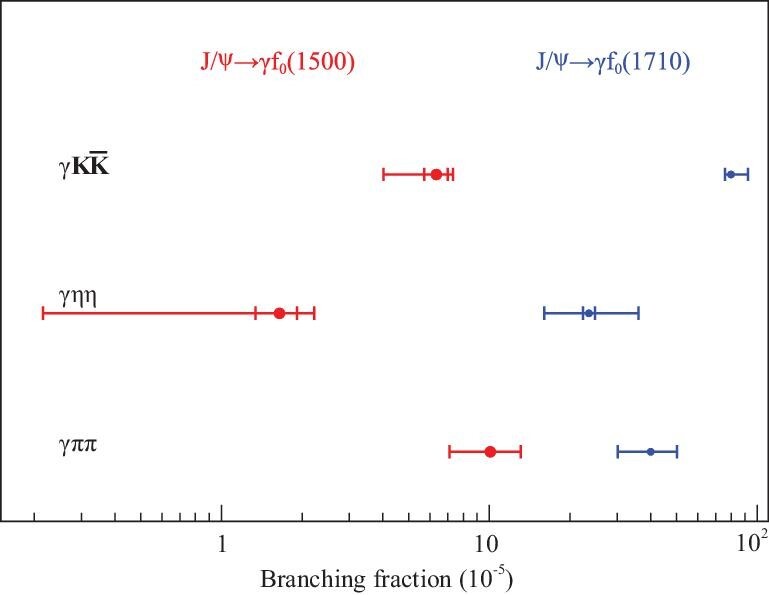
The comparison of the product branching fractions in different processes.

We show a comparison between the product branching fractions for the scalar glueball candidates *f*_0_(1500) and *f*_0_(1710) in different decay modes in Fig. 5.

By taking ${\cal B}(f_0(1500) \rightarrow \pi \pi ) = (34.9 \pm 2.3)\%$ and ${\cal B}(f_0(1710) \rightarrow K \bar{K}) = (36.0\pm 12.0)\%$ from the PDG tables, together with BES product branching fraction results, we determine the *f*_0_(1500) and *f*_0_(1710) production rates in *J*/ψ radiative decays. A comparison of the measured production rates with those obtained from LQCD calculations for a scalar glueball is given in Table 2.

**Table 2.** The production rates of *f*_0_(1500) and *f*_0_(1710) in *J*/ψ radiative decays from experiments and LQCD calculations [66].

		
		${\cal B}(J/\psi \rightarrow \gamma$ scalar glueball)
${\cal B}(J/\psi \rightarrow \gamma f_0(1500))$	${\cal B}(J/\psi \rightarrow \gamma f_0(1710))$	(LQCD calculation)
(10^−3^)	(10^−3^)	(10^−3^)
∼0.29	∼2.2	3.8 (8)

The production rate for *f*_0_(1710) in gluon-rich *J*/ψ radiative decays is close to LQCD calculations for a scalar glueball and is about an order of magnitude larger than that for *f*_0_(1500). This might suggest that *f*_0_(1710) has a larger gluonic component than *f*_0_(1500). Studies of *f*_0_(1500) and *f*_0_(1710) production in other gluon-favored and gluon-disfavored processes will be crucial to conclusively establish the scalar glueball. For the *f*_2_(2340) tensor state, the LQCD prediction for the production rate of a pure-gauge tensor glueball in radiative *J*/ψ decays  [67] is Γ_*TensorGlueball*_/Γ_*total*_ = 1.1(2) × 10^−2^. The presence of *f*_2_(2340) in the ηη [63], *K*_*S*_*K*_*S*_ [64] and φφ [65] final states suggests that *f*_2_(2340) might be a candidate for the tensor glueball. However, the current measured production rate for *f*_2_(2340), based on the observed ηη, $K \bar{K}$ and φφ modes alone, appears to be substantially lower than that obtained in the LQCD calculation. Searches for additional decay modes of *f*_2_(2340) are needed.

### Pseudoscalar states

The ground states of the *I* = 0, *J*^*PC*^ = 0^−+^ pseudoscalars are the η and η^′^. The small number of expected radial excitations for 0^−+^ states in the quark model provides a clean and promising environment for the search of pseudoscalar glueballs.

#### η(1405/1475)

A pseudoscalar state around 1440 MeV/c^2^, η(1440), was first observed in $p\bar{p}$ annihilation at rest into η(1440)π^+^π^−^ with η(1440) → ηπ^+^π^−^ and $K \bar{K} \pi$ [68], and further observed in the π^−^*p* process [69,70] and *J*/ψ radiative decays [71,72]. Considerable theoretical and experimental efforts have been made to try to understand its nature. It was proposed as a candidate for a pseudoscalar glueball [73,74], due to its copious production in gluon-rich processes. However, the measured mass is much lower than that obtained from lattice QCD calculations, which is above 2.3 GeV/c^2^ [31]. Subsequent experiments produced evidence that this state was really two different pseudoscalar states, η(1405) and η(1475). The former has large couplings to *a*_0_(980)π or direct $K\bar{K}\pi$, while the latter decays mainly to $K^{*}(892)\bar{K}$. A detailed review of the experimental situation can be found in the review by PDG2020 for pseudoscalar and pseudovector mesons in the 1400 MeV region [16] or in [75]. However, it remains controversial whether one or two pseudoscalar mesons exist in this mass region. Klempt *et al.* [76] claimed that the splitting of a single state could be due to nodes in the decay amplitudes that differ for the ηππ and $K^*(892) \bar{K}$ channels.

**Figure 6.**
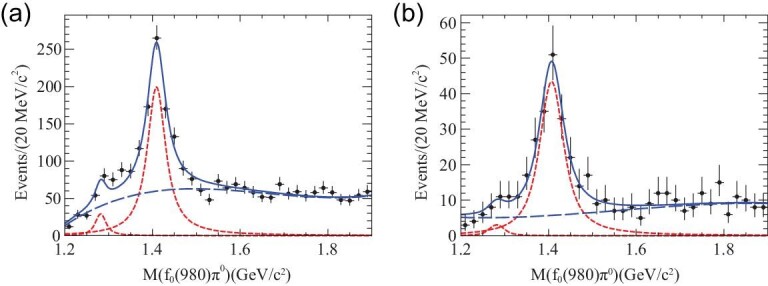
Results of the fit to (a) the *f*_0_(980)(π^+^π^−^)π^0^ and (b) *f*_0_(980)(π^0^π^0^)π^0^ invariant mass spectra.

With 2.25 × 10^8^ *J*/ψ events collected with the BESIII detector, the decays of *J*/ψ → γπ^+^π^−^π^0^ and γ3π^0^ were studied [77]. The isospin-violating decay η(1405) → *f*_0_(980)π^0^ was observed for the first time with a statistical significance larger than 10σ in both the charged (*f*_0_(980) → π^+^π^−^, Fig. 6(a)) and neutral (*f*_0_(980) → π^0^π^0^, Fig. 6(b)) modes. The isospin violating ratio ${\cal B}(\eta (1405)\rightarrow f_{0}(980)\pi ^{0} \rightarrow \pi ^{+}\pi ^{-}\pi ^{0})$ to ${\cal B}(\eta (1405)\rightarrow a_{0}(980)\pi ^{0} \rightarrow \eta \pi ^{+}\pi ^{-})$ is $(17.9\pm 4.2)\%$ [16,77,78], which is an order of magnitude larger than the $a_0^{0}(980)-f_0(980)$ mixing intensity (less than 1%) that was measured by BESIII [79].

The anomalous large isospin violations in *J*/ψ → γη(1405/1475) → γπ^0^*f*_0_(980) → γ3π stimulated many theoretical efforts to understand the nature of η(1405/1475). With the assumption that only one 0^−+^ exists around 1.4 GeV/c^2^, the triangle singularity mechanism was found to play a more dominant role than *a*_0_(980) − *f*_0_(980) mixing, and it can produce the anomalously large isospin violations in η(1405) → π^+^π^−^π^0^, according to [80,81].

The η(1405/1475) state was also observed in *J*/ψ decays to γη(1405/1475) and η(1405/1475) → γφ, with 1.3 × 10^9^ *J*/ψ events at BESIII  [82]. The observation of η(1405/1475) → γφ indicates that η(1405/1475) contains a sizable $s \bar{s}$ component and this does not match very well to the expectations for a pseudoscalar glueball.

#### *X*(2370)

**Figure 7.**
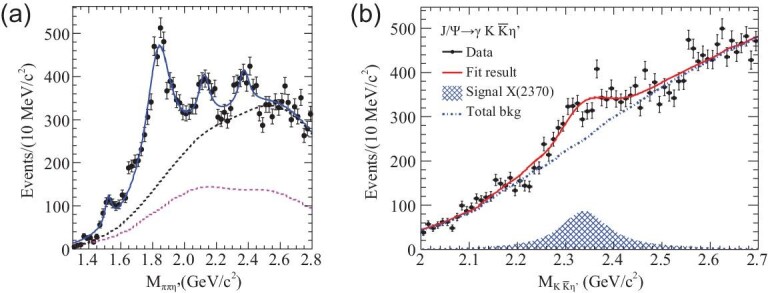
Results of the fit to (a) the η^′^π^+^π^−^ and (b) $\eta ^{\prime } K \bar{K}$ invariant mass spectra.

The mass for the lightest pseudoscalar glueball is expected to be higher than 2.3 GeV/c^2^ from LQCD calculations, while the existence of any pseudoscalar states above 2.0 GeV/c^2^ is not well established experimentally. In *J*/ψ → γη^′^π^+^π^−^ decays at BESIII [83], the observation of *X*(1835) by BESII [84] was confirmed, as is shown in Fig. 7(a) (to be discussed in detail in the next section) in the η^′^π^+^π^−^ invariant mass distribution. In addition, two additional states, *X*(2120) and *X*(2370), are observed with statistical significances larger than 7.2σ and 6.4σ, respectively. The mass of the *X*(2370) state is measured to be $M= 2376.3 \pm 8.7 ^{+3.2} _{-4.3}$ MeV/*c*^2^ from a one-dimensional fit. The *X*(2370) state has been further confirmed in the $\eta ^{\prime }K \bar{K}$ invariant mass distribution in *J*/ψ radiative decays (shown in Fig. 7 (b)) [85] with a statistical significance of 8.3σ. The fitted masses of *X*(2370) in the two decay modes agree with each other, and coincide with the mass of the lightest pseudoscalar glueball from LQCD calculations, which makes *X*(2370) a candidate for the lightest pseudoscalar glueball. However, it is crucial to determine its spin parity and observe it in more decay modes before this conclusion can be firmly established.

## SEARCH FOR HYBRID STATES WITH EXOTIC QUANTUM NUMBERS

Hybrid states are color-singlet combinations of constituent quarks and gluons, such as a $q \bar{q} g$ state. Evidence for the existence of hybrid states would be direct proof of the existence of gluonic degrees of freedom in hadrons. Low mass hybrids have the additional attraction that, unlike low-lying glueballs, they could have exotic *J*^*PC*^ quantum numbers, in which case they would not mix with conventional $q \bar{q}$ states. This exotic quantum number signature for hybrid states allows for the unambiguous identification of hybrids.

**Figure 8.**
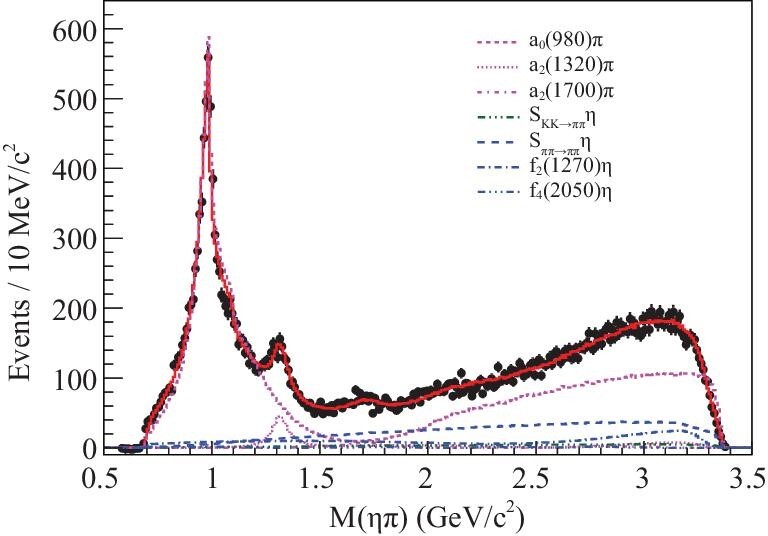
The ηπ invariant mass, compared with amplitude analysis fit (solid curve) with corresponding amplitudes (various dashed and dotted lines).

The observation of isovector 1^−+^ exotic hybrid candidates, i.e. π_1_(1400) and π_1_(1600), which decay into different final states, such as ηπ, η^′^π, *f*_1_(1285)π, *b*_1_(1235)π and ρπ, were reported in different reactions. The evidence for π_1_(2015) has also been reported. Reviews of the experimental status on these isovector 1^−+^ exotic states can be found in [16,76,86–88]. With 4.48 × 10^8^ ψ(3686) events collected with BESIII, an amplitude analysis is applied to ψ(3686) → γχ_*c*1_, χ_*c*1_ → ηπ^+^π^−^ to search for π_1_(1400), π_1_(1600) and π_1_(2015) [89]. Figure 8 shows the ηπ invariant mass, compared with results of an amplitude analysis fit (solid curve) with various corresponding amplitudes (dashed and dotted lines). There is no significant 1^−+^ state in the ηπ invariant mass spectrum, and upper limits for the branching fractions χ_*c*1_ → π_1_(1400)^±^π^∓^, χ_*c*1_ → π_1_(1600)^±^π^∓^ and χ_*c*1_ → π_1_(2015)^±^π^∓^, with subsequent π_1_(*X*)^±^ → ηπ^±^ decay, are established. BESIII searches for isovector exotic states in η^′^π invariant mass spectra are ongoing.

There is no evidence for the existence of isosinglet 1^−+^ states. The theoretical predictions for their main decay modes are *f*_1_(1285)η, *a*_1_π and ηη^′^, etc. [90–93]. The 10 billion *J*/ψ events that were recently accumulated by BESIII provide an ideal laboratory for the search for such states.

## NEW HADRONS NEAR THE PROTON-ANTIPROTON MASS THRESHOLD

**Figure 9.**
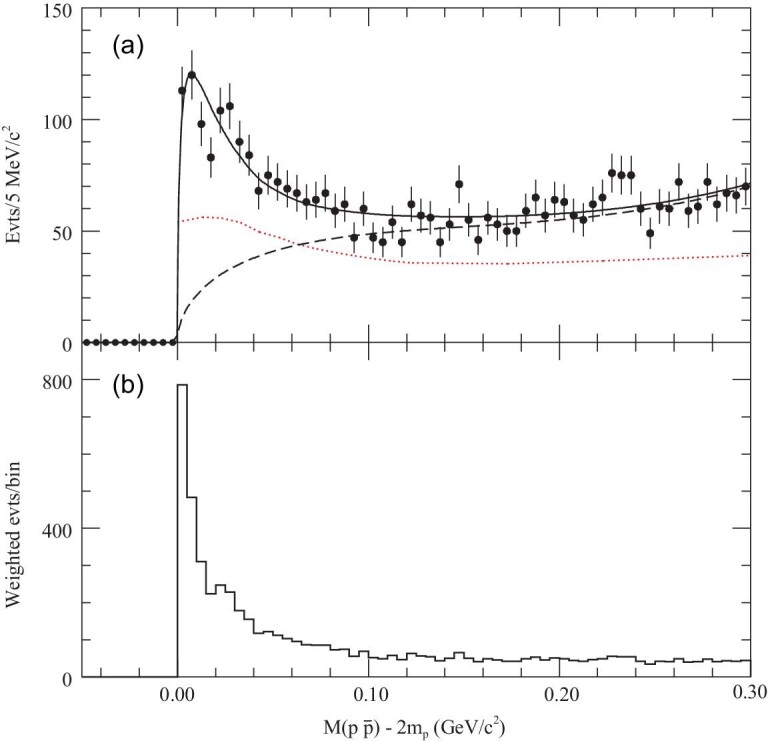
(a) The near threshold $M_{p\bar{p}}-2m_{p}$ distribution. The solid curve is the result of the fit; the dashed curve shows the fitted background function. The dotted curve indicates how the acceptance varies with $p \bar{p}$ invariant mass. (b) The $M_{p\bar{p}}-2m_{p}$ distribution with phase space correction.

An anomalously proton-antiproton ($p\bar{p}$) mass threshold enhancement, $X(p\bar{p})$, was first observed by BESII in $J/\psi \rightarrow \gamma p\bar{p}$ decays [94] (Fig. 9) and later confirmed by BESIII [95] and CLEO [96]. This strong enhancement was subsequently determined to have spin parity *J*^*P*^ = 0^−^ by BESIII [97], with a mass of ${M=1832}\, {}^{+19}_{-5}\, (\text{ stat.})\, {}^{+18}_{-17}\, ({\text{syst.}}){\pm 19}\, ({\text{model}})$ MeV/c^2^ and width of $ \Gamma <76\, \rm MeV $/c^2^ at the 90$\%$ C.L. The non-observation of $X(p\bar{p})$ in $ J/\psi \rightarrow \omega p\bar{p} $ indicates that the pure final-state interaction (FSI) interpretation is disfavored for this structure [98]; however, FSI effects should be included in the fit of the $p\bar{p}$ mass spectrum near threshold and they have significant impact on the parameters of the $X(p\bar{p})$ resonance [97].

**Figure 10.**
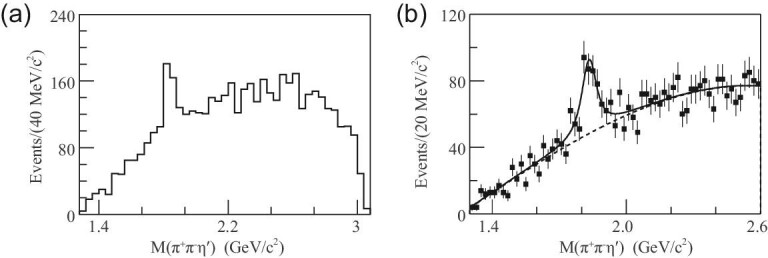
(a) and (b) The η^′^π^+^π^−^ invariant mass distribution. The figure (b) shows the fit (solid curve) to the data (points with error bars); the dashed curve indicates the background function.

**Figure 11.**
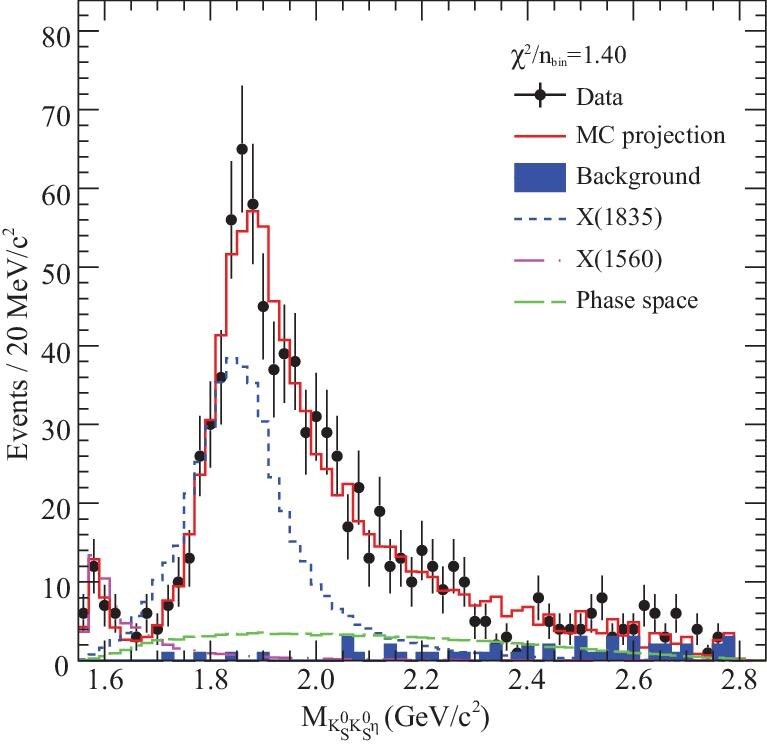
Invariant mass distribution of $K^{0}_{S} K^{0}_{S}\eta$. The black dots with error bars represent data, the red histograms are the PWA projections and the blue shaded histograms show backgrounds estimated by the η sideband. The contribution of *X*(1835) is shown by the blue short-dashed histograms.

The *X*(1835) state was first observed by the BESII experiment as a peak in the η^′^π^+^π^−^ invariant mass distribution in *J*/ψ → γη^′^π^+^π^−^ decays [84] (Fig. 10). It was later confirmed by BESIII studies of the same process [83] (Fig. 7) with mass and width measured to be $M=1836.5\pm 3\, ^{+5.6}_{-2.1}\, \rm MeV$/c^2^ and $\Gamma =190\pm 9\, ^{+38}_{-36}\, \rm MeV$/c^2^; the *X*(1835) state was also observed in the $K^{0}_{S} K^{0}_{S}\eta$ invariant mass spectrum in $J/\psi \rightarrow \gamma K^{0}_{S}K^{0}_{S}\eta$ decays (Fig. 11), where its spin parity was determined to be *J*^*P*^ = 0^−^ by a model-dependent PWA [99]. A new decay mode of *X*(1835) decaying into γφ was recently observed in *J*/ψ → γγφ [82].

One of the theoretical interpretations of the natures of *X*(1835) and $X(p\bar{p})$ [100–105] suggests that the two structures originate from a $p\bar{p}$ bound state [106–110]. If *X*(1835) is really a $p\bar{p}$ bound state, it should have a strong coupling to 0^−^$p\bar{p}$ systems, in which case the line shape of *X*(1835) at the $p\bar{p}$ mass threshold would not be described as a simple BW line shape. A study of the η^′^π^+^π^−^ line shape of *X*(1835) with high statistical precision therefore provides valuable information that helps clarify the natures of *X*(1835) and $X(p\bar{p})$.

With 1.09 × 10^9^ *J*/ψ events accumulated at the BESIII experiment, we studied the *J*/ψ → γη^′^π^+^π^−^ process and observed a significant abrupt change in the slope of the η^′^π^+^π^−^ invariant mass distribution at the proton-antiproton ($p\bar{p}$) mass threshold. Two models were used to characterize the η^′^π^+^π^−^ line shape around 1.85 GeV/c^2^: one explicitly incorporates the opening of a decay threshold in the mass spectrum (Flatté formula), and another is the coherent sum of two resonant amplitudes.

In the first model, we assume that state *X*(1835) couples to $p\bar{p}$. The line shape of η^′^π^+^π^−^ above the $p\bar{p}$ threshold is therefore affected by the opening of the $X(1835)\rightarrow p\bar{p}$ decay channel, similar to the distortion of the *f*_0_(980) → π^+^π^−^ line shape at the $K\bar{K}$ threshold. To study this, the Flatté formula [111], defined below, is used to describe the *X*(1835) line shape:

(1) \begin{equation*} T=\frac{\sqrt{\rho _{\rm out}}}{\mathcal {M}^{2}-s-i\sum _{k}g^{2}_{k}\rho _{k}}. \end{equation*}

Here, *T* is the decay amplitude, ρ_out_ is the phase space for *J*/ψ → γη^′^π^+^π^−^, $\mathcal {M}$ is a parameter with the dimension of mass, *s* is the square of the η^′^π^+^π^−^ system mass, ρ_*k*_ is the phase space for decay mode *k* and $g^{2}_{k}$ is the corresponding coupling strength. The term $\sum _{k}g^{2}_{k}\rho _{k}$ describes how the decay width varies with *s*:

(2) \begin{equation*} { \sum _{k}g^{2}_{k}\rho _{k}\approx g^{2}_{0}\bigg (\rho _{0}+\frac{g^{2}_{p\bar{p}}}{g^{2}_{0}}\rho _{p\bar{p}}\bigg ). } \end{equation*}

Here, $g^{2}_{0}$ is the sum of *g*^2^ of all decay modes other than $X(1835)\rightarrow p\bar{p}$, ρ_0_ is the maximum two-body decay phase space volume [16] and $g^{2}_{p\bar{p}}/g^{2}_{0}$ is the ratio between the coupling strength to the $p\bar{p}$ channel and the sum of all other channels.

**Figure 12.**
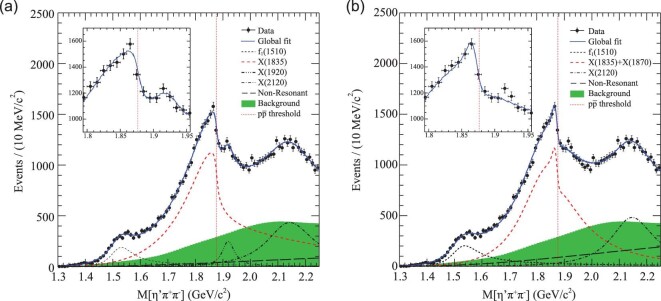
(a) Fit results using the Flatté formula. (b) Fit results based on a coherent sum of two Breit–Wigner amplitudes. The dash–dot vertical lines show the position of the $p\bar{p}$ mass threshold, the black dots with error bars represent data, the solid curves are total fit results, the red dashed curve in (a) shows the state around $1.85\, \rm GeV$/c^2^ and the red dashed curve in (b) is the sum of *X*(1835) and *X*(1870). The insets show the data and global fit between 1.8 and $1.95\, \rm GeV$/c^2^.

The fit results for this model are shown in Fig. 12(a). The fit yields $g^{2}_{p\bar{p}}/g^{2}_{0}=2.31\pm 0.37\, ^{+0.83}_{-0.60}$ with a statistical significance of $g^{2}_{p\bar{p}}/g^{2}_{0}$ being non-zero larger than 7σ. The value of $g^{2}_{p\bar{p}}/g^{2}_{0}$ implies that the couplings between the *X*(1835) and $X(p\bar{p})$ final states is very large. According to the definitions given in [112], the pole position is determined by requiring that the denominator in Equation (1) be zero. The pole that is nearest to the $p\bar{p}$ mass threshold is found to be $M_{\rm pole}=1909.5\pm 15.9\, ({\rm stat.})\, ^{+9.4}_{-27.5}({\text{syst.}})\, \rm MeV$/c^2^ and $\Gamma _{\rm pole}=273.5\pm 21.4\, ({\rm stat.})\, ^{+6.1}_{-64.0}\, ({\rm syst.})\, \rm MeV$/c^2^.

In the second model, we assume that the distortion comes from the interference between *X*(1835) and another resonance with mass close to the $p\bar{p}$ mass threshold. A fit with a coherent sum of two interfering Breit–Wigner amplitudes to describe the η^′^π^+^π^−^ mass spectrum around $1.85\, \rm GeV$/c^2^ is performed. This fit yields a narrow resonance below the $p\bar{p}$ mass threshold with $M=1870.2\pm 2.2(\text{stat.})^{+2.3}_{-0.7}(\text{syst.})\, \rm MeV$/c^2^ and $\Gamma =13.0\pm 6.1(\text{stat.})^{+2.1}_{-3.8}(\text{syst.})\, \rm MeV$/c^2^, with a statistical significance larger than 7σ. The fit results for the second model are shown in Fig. 12(b).

Based on current data samples, two models fit the data with similar fit qualities. Both fits suggest the existence of either a broad state with strong couplings to $p\bar{p}$, or a narrow state just below the $p\bar{p}$ mass threshold. For the former case, its strong coupling to $p\bar{p}$ suggests the existence of a $p\bar{p}$ molecule-like state. For the latter case, the narrow state just below the $p\bar{p}$ mass threshold suggests that it is an unconventional meson, possibly a $p\bar{p}$ bound state. So both fits support the existence of a $p\bar{p}$ molecule-like or bound state. However, more sophisticated models such as a mixture of the above two models cannot be ruled out.

## SUMMARY AND PERSPECTIVES

Continuous experimental efforts are being made to search for and study glueballs, hybrids and multi-quark states from charmonium decays, supported by the huge statistics data samples accumulated at BESIII.

We have found that the production rate for *f*_0_(1710) in gluon-rich *J*/ψ radiative decays is about an order of magnitude higher than that for *f*_0_(1500) and is close to LQCD calculations for the production rate of a scalar glueball, under current circumstance. This suggests that *f*_0_(1710) can have a larger gluonic component than *f*_0_(1500). Studies of *f*_0_(1500) and *f*_0_(1710) in other gluon-favored and gluon-disfavored processes with improved analysis techniques will be crucial to further refine this conclusion. The mass of the *f*_2_(2340) tensor state matches the LQCD expectation for a pure tensor glueball. This, and its copious production in *J*/ψ radiative decays to ηη, *K*_*S*_*K*_*S*_ and φφ, might suggest that *f*_2_(2340) is a candidate of the tensor glueball. However, the current measured production rates for *f*_2_(2340) appear to be substantially lower than LQCD expectations. Since no dominant glueball decay mode can be expected, due to the flavor blindness of glueball decays, searches for additional *f*_2_(2340) decay modes are necessary. In light of the observation of *X*(2370) in *J*/ψ → γη^′^π^+^π^−^ and $\gamma \eta ^{\prime } K \bar{K}$, the identification of the lowest-pseudoscalar glueball has become a recent major focus of BESIII. In particular, the 10 billion *J*/ψ event sample and the clean environment in *J*/ψ → γη^′^*K*_*S*_*K*_*S*_ decays will make an amplitude analysis and the determination of the spin parity of *X*(2370) possible.

In searching for hybrid states with exotic quantum numbers, no significant signals for the isovector 1^−+^ exotic hybrid candidates π_1_(1400), π_1_(1600) and π_1_(2015) were seen in the ψ(3686) → γχ_*c*1_, χ_*c*1_ → ηπ^+^π^−^ decay process with 4.48 × 10^8^ ψ(3686) events collected with BESIII. As of yet, no evidence for an isoscalar 1^−+^ exotic hybrid has been found. The 10 billion *J*/ψ event sample will provide an ideal laboratory for the search of isoscalar 1^−+^ exotic hybrids in *f*_1_(1285)η, *a*_1_π and ηη^′^, etc. decay channels.

In order to elucidate further the nature of the states around $1.85\, \rm GeV$/c^2^, more data are needed to further study the *J*/ψ → γη^′^π^+^π^−^ process. Also, line shapes for other radiative decay channels should be studied near the $p\bar{p}$ mass threshold, along with further studies of $J/\psi \rightarrow \gamma p\bar{p}$ and $J/\psi \rightarrow \gamma \eta K^{0}_{S}K^{0}_{S}$.
